# A Graphene-Based Glycan Biosensor for Electrochemical Label-Free Detection of a Tumor-Associated Antibody

**DOI:** 10.3390/s19245409

**Published:** 2019-12-09

**Authors:** Filip Kveton, Anna Blsakova, Lenka Lorencova, Monika Jerigova, Dusan Velic, Ola Blixt, Bo Jansson, Peter Kasak, Jan Tkac

**Affiliations:** 1 Department of Glycobiotechnology, Institute of Chemistry, Slovak Academy of Sciences, Dubravska cesta 9, 845 38 Bratislava, Slovakia; filipkvietok@gmail.com (F.K.); chemabls@savba.sk (A.B.); lenka.lorencova@savba.sk (L.L.); 2 Department of Physical Chemistry, Faculty of Natural Sciences, Comenius University, Mlynska Dolina, 842 15 Bratislava, Slovakia; jerigova@ilc.sk (M.J.); duvellabs@gmail.com (D.V.); 3 International Laser Centre, Ilkovicova 3, 841 04 Bratislava, Slovakia; 4 Department of Chemistry, University of Copenhagen, 1871 Copenhagen, Denmark; olablixt@chem.ku.dk; 5 Division of Oncology and Pathology, Department of Clinical Sciences, Lund, Lund University, Lund, SE 221 85, Sweden; bo.jansson@med.lu.se; 6 Center for Advanced Materials, Qatar University, P.O. Box 2713, Doha, Qatar

**Keywords:** graphene screen-printed electrodes, Tn antigen, glycan, biosensor, electrochemistry

## Abstract

The study describes development of a glycan biosensor for detection of a tumor-associated antibody. The glycan biosensor is built on an electrochemically activated/oxidized graphene screen-printed electrode (GSPE). Oxygen functionalities were subsequently applied for covalent immobilization of human serum albumin (HSA) as a natural nanoscaffold for covalent immobilization of Thomsen-nouvelle (Tn) antigen (GalNAc-*O*-Ser/Thr) to be fully available for affinity interaction with its analyte—a tumor-associated antibody. The step by step building process of glycan biosensor development was comprehensively characterized using a battery of techniques (scanning electron microscopy, atomic force microscopy, contact angle measurements, secondary ion mass spectrometry, surface plasmon resonance, Raman and energy-dispersive X-ray spectroscopy). Results suggest that electrochemical oxidation of graphene SPE preferentially oxidizes only the surface of graphene flakes within the graphene SPE. Optimization studies revealed the following optimal parameters: activation potential of +1.5 V *vs.* Ag/AgCl/3 M KCl, activation time of 60 s and concentration of HSA of 0.1 g L^−1^. Finally, the glycan biosensor was built up able to selectively and sensitively detect its analyte down to low aM concentration. The binding preference of the glycan biosensor was in an agreement with independent surface plasmon resonance analysis.

## 1. Introduction

The surface of every living cell is decorated with a compact layer of complex carbohydrates (glycans) adhered to a cell membrane. Thus, for an elementary understanding of biology it is important to get a detailed knowledge about the functions of glycans [1,2]. Over 50% of all proteins in organisms are modified by glycosylation and such a post-translational modification is crucial for regulation of many cellular processes. Glycans are built-up step-by-step using an enzymatic addition of carbohydrate building blocks to proteins/lipids [3]. Aberrant glycosylation is associated with many cellular properties involving cell proliferation, differentiation, transformation, migration, invasion, apoptosis, and immune responses.

Cancer is one of the predominant diseases leading to mortality, with approximately 14.1 million new malignant cases with 8.2 million associated deaths every year [4]. Due to the progressive increase of the disease occurrence we can speak of a ’cancer epidemic’ [5]. Cancer cells undergo significant modifications in terms of glycan expression. Glycosylation patterns and level of glycans on the cell surfaces often give us information about the biological condition of the cells. Various tumor-associated carbohydrate antigens (TACAs) were identified to mediate key steps during cancer progression. For example TACAs such as the Thomsen-nouvelle (Tn; GalNAc-*O*-Ser/Thr), sTn (sialyl Tn; NeuAcα2-6GalNAcα-*O*-Ser/Thr), and Thomsen-Friedenreich (TF; Galβl-3GalNAcαl-*O*-Ser/Thr) [6,7] antigens can be used as diagnostic tumor markers and therapeutic targets [8]. The TF antigen is over-expressed in many carcinoma cells and it is biosynthesized from the Tn core. Correlation between levels of the TF and the Tn has never been observed [9].

However, the most common and very specific TACA is the Tn antigen discovered in 1957 [10]. The Tn antigen as a small glycan is expressed early in transformed cells and is the precursor for synthesis of other aberrant *O*-glycans [11]. Its presence was confirmed in 70–90% of cancer types [11,12,13,14,15,16] and its expression corresponds with metastatic progression of the disease and a poor prognosis for cancer patients. On the other hand, good prognosis for cancer patients is associated with high levels of naturally generated anti-TACA antibodies (antibodies against aberrant glycans) [17]. 

Early cancer detection and personalized patient treatment are required in order to improve the survival rates of cancer patients [18]. Thus, cost-effective and simple tests and methods, which can detect cancer biomarkers in a mildly invasive or non-invasive way directly in serum/urine are required. Conventional instrumental-based methods have distinct drawbacks, when applied in glycomics and for diagnostic purposes [19], this is why affinity-based devices have a huge potential in cancer diagnostics [20]. Devices based on electrochemical methods offer ultrasensitive, rapid, simple, reliable, and economical assay protocols applicable also for biomarkers detection [21,22,23].

Nanotechnology is a driving force for advancements in many scientific disciplines including also cancer diagnostics, cancer therapy and glycomics [24,25]. Nanoparticles and nanomaterials with engineered surface characteristics can be effectively applied as scaffolds for displaying of glycans that allow precise surface positioning and higher density than using traditional approaches [22]. Furthermore, addition of nanomaterials such as NPs, carbon nanotubes (CNTs) or graphene may enhance sensitivity of biosensing [26,27]. The most prominent nanomaterial is graphene with unique physico-chemical characteristics. One-atom 2D thick layer of *sp*^2^ hybridized carbon atoms—graphene — has fascinated the scientific community since the early description of its properties in 2004 [28,29]. Despite their short history, graphene-based materials are frequently used in (electro)biosensing thanks to their exceptional, unique and impressive electrical, thermal, optical and mechanical properties as a consequence of its configuration [30,31]. Moreover, depending on the purpose, graphene surface can be easily functionalized through non-covalent (π-π stacking, hydrophobic and electrostatic interactions) or covalent interaction (utilization of free oxygen groups in graphene oxide). Our recent study suggests [32] that unmodified graphene due to hydrophobic nature can effective denature proteins, that can result in development of interfacial layers exhibiting substantially compromised bioreceptive properties and/or being significantly prone to non-specific protein binding, while working with complex samples like human serum. 

In our recent review paper we found out that several glycan biosensors developed on graphene modified interfaces offer quite high limits of detection (i.e., in the range of nM–μM) (see also Table 1 in the current manuscript) [22]. In this manuscript we wanted to investigate if such biosensing performance is the attribute of the detection platform used (FET sensing or electrochemistry using built-in redox mediators) or it is an inherent attribute of graphene interface. This is why in this study we investigate basic characteristics and biosensing performance of graphene screen-printed electrodes (GSPE) oxidized by potentiostatic activation to design glycan biosensor for detection of a tumor-associated antibody.

## 2. Materials and Methods

### 2.1. Chemicals

Potassium hexacyanoferrate (II) trihydrate, *N*-hydroxysuccinimide (NHS), *N*-(3-dimethylaminopropyl)-*N*′-ethylcarbodiimide hydrochloride (EDC), phosphate buffered saline tablet (PBS, one tablet dissolved in 200 mL of deionized water (DW) yields 0.01 M phosphate buffer, 0.0027 M potassium chloride and 0.137 M sodium chloride, pH 7.4, at 25 °C), KH_2_PO_4_, K_2_HPO_4_, TWEEN 20, lectin from *Dolichosbiflorus* (DBA), Concanavalin A (Con A) and human serum albumin (HSA), were of ≥99% purity or p.a. grade and were purchased from Sigma Aldrich (St. Louis, MO, USA). 

Antibody GOD3-2C4 (2C4), a mouse IgG1κ antibody not cross-reacting with the GalNAc-β-*O* epitope or the blood group A antigen and specific to both synthetic Tn antigens and mucin-associated Tn antigen, was produced using a procedure as published by Jansson and co-workers [33]. Antibody GOD3-2C4 binds to the Tn antigen expressed by cancer of breast, colon, lung, ovary, and pancreas and was the first anti-Tn antibody showing anti-tumor activity on a solid tumor *in-vivo* [33] and recently the antibody was applied to identify possible carrier of the Tn antigen in samples from patients having breast cancer [15]. Binding specificity towards various glycans, glycoprotein and proteins showed no binding of GOD3-2C4 antibody to BSA or HSA proteins with a biospecific binding towards the Tn antigen [33]. The Tn antigen (GalNAcα1-*O*-serine) was provided from Carbosynth (Newbury, UK). The Tn-BSA (Tn antigen conjugated to bovine serum albumin) conjugate for Surface Plasmon Resonance (SPR) experiments was purchased from GLYcoDiag (Orléans, France).

All solutions and buffer components were freshly prepared in 0.055 μS cm^−1^ ultrapure deionized water (DW) and filtered *prior* to use using 0.2 μm sterile filters. HSA was dissolved in 10 mM PBS solution with pH 7.4 and 0.05 % TWEEN 20. The Tn antigen was dissolved in 10 mM PBS solution with pH 7.4, both solutions were prepared at concentration of 1 mg mL^−1^ and were stored at −20 °C in aliquots.

### 2.2. Electrode Pretreatment

First, the surfaces of bare graphene screen-printed electrodes (GSPEs, *d* = 4 mm, DropSens, Llanera, Spain) were potentiostatically activated. Chronoamperometry was chosen as an activation procedure. We started with optimization of an activation time and potential. Three different time intervals (30 s, 60 s and 90 s) in combination with two different potential values (+1.5 V and +1.7 V) were examined [34]. The process was carried out in three-electrode electrochemical cells with an Ag/AgCl/3 M KCl reference and a counter Pt electrode (Bioanalytical Systems, West Laffayette, IN, USA) using phosphate buffer (50 mM, pH 6.0). The actual measurement was carried out by a laboratory potentiostat/galvanostatAutolab PGSTAT 302N (Ecochemie, Utrecht, The Netherlands). Measurements were run under Nova Software 1.10.

### 2.3. The Glycan Biosensor

After electrochemical activation step, working surfaces of GSPEs were washed with DW. Free (electro)activated carboxyl groups were activated with 40 µL solution of 200 mM EDC and 50 mM NHS mixed at a ratio of 1+1 just *prior* immobilization (solution of EDC and NHS were previously prepared in DW and stored separately at −80 °C in aliquots) for 12 min [35]. After this chemical activation, the electrodes were washed with DW. The next step was an incubation of surfaces with HSA (10^−5^−10^−1^ mg mL^−1^ dissolved in PBS with 0.05% TWEEN 20) for 15 min. After immobilization of HSA, the protein was activated with 40 µL solution of 200 mM EDC and 50 mM NHS at a ratio of 1 + 1 for 12 min and then the activated surface was incubated with the Tn antigen (100 µM) for 15 min. The HSA and glycan immobilization were performed at a room temperature.

### 2.4. Differential Pulse Voltammetry (DPV) Measurement

**Figure 1:**
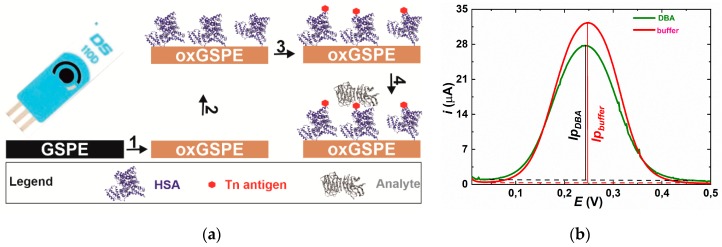
(**a**) Modification of graphene screen-printed electrode (GSPE) by electrochemical oxidation (step 1), covalent immobilization of human serum albumin (HSA) as a natural nanoscaffold (step 2) and a covalent immobilization of a Tn antigen to HSA (step 3); The final step is incubation with the analyte protein (step 4); (**b**) Typical DPV obtained on the electrode incubated with plain buffer (red) and after incubation with 9×10^−12^ M DBA lectin. Relative response towards DBA is calculated as Δ*I*=(*I*_pbuffer_−*I*p_DBA_)/*I*p_buffer_×100.

DPV was measured in an electrolyte containing 5 mM potassium hexacyanoferrate (II) trihydrate and 0.01 M PBS, pH 7.4. The parameters applied for the differential pulse voltammetry were as follows: 60 s accumulation time at 0.2 V, 50 ms modulation time, 0.5 s interval time, 25 mV modulation amplitude, and 5 mV step. Measurements were run under Nova Software 1.10 (Ecochemie,). The results were presented in a form *i* vs. *E* plot where a peak height was compared and analyzed (Figure 1b) for analyte (lectin or GOD3-2C4 antibody) concentration typically from 9 aM up to 9 pM. The biosensor exhibits saturation of the response signal at concentrations higher than 9 pM (Figure S1). Each analyte was measured at least in triplicate on three independent biosensor devices (electrodes) and results are shown with a standard deviation (±SD) or relative standard deviation (RSD) calculated in Excel. It is worth noting that such RSDs are not relative standard errors of analyte detection, but rather represent reproducibility of the biosensor construction, since each calibration curve was constructed by an independent biosensor device. Measurements of a particular analyte were performed on the same day. See the Electronic Supporting Material (ESM) file for other characterization tools applied in the study.

## 3. Results and Discussion

The glycan biosensor was developed in three consecutive steps: 1. Activation of GSPE by an anodic potential; 2. Covalent immobilization of HSA on activated surface of GSPE electrode and 3. Covalent attachment of Tn antigen on HSA layer (Figure 1a).

### 3.1. Optimization of Construction of the Glycan Biosensor 

#### 3.1.1. Electrode Activation

The first parameter, which was optimized was the process of electrode surface activation by application of an anodic potential (+1.5 V and +1.7 V *vs.* Ag/AgCl/3 M KCl) for defined time (30 s, 60 s and 90 s). There are two reasons for oxidation of graphene surface. The first one is deposition of oxygenated functional groups like -COOH for covalent attachment of HSA as a natural nanoscaffold for subsequent immobilization of the Tn antigen. The second reason is to prevent denaturation of HSA after interaction with graphene surface and to prevent exposure of hydrophobic parts of the protein backbone after denaturation, what can induce non-specific binding of proteins from complex matrices [32]. 

**Figure 2:**
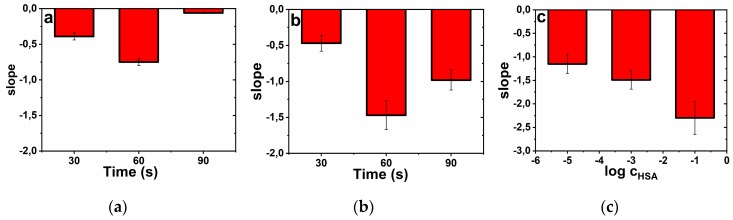
Optimization of the glycan biosensor preparation. (**a**) Application of an anodic potential of +1.5 V *vs.* Ag/AgCl/3 M KCl or (**b**) of +1.7 V *vs.* Ag/AgCl/3 M KCl to activate graphene SPE and (**c**) optimization of the effect of HSA concentration (expressed in g L^−1^) on the glycan biosensor sensitivity towards DBA lectin as an analyte.

The results suggest that at an oxidation potential of +1.5 V *vs.* Ag/AgCl/3 M KCl, the highest sensitivity of −(0.75 ± 0.05) % M^−1^ for DBA lectin detection was observed after application of the potential for 60 s (Figure 2a). When the GSPE was activated by the anodic potential of +1.7 V *vs.* Ag/AgCl/3 M KCl, again the optimal time of oxidation was 60 s with the sensitivity of the DBA detection of −(1.47 ± 0.2) % M^−1^ (Figure 2b), representing an increase of the biosensor sensitivity 2-fold. In order to prepare the glycan biosensor in a robust way, for further work an activation potential of +1.5 V applied for 60 s was selected since the biosensor based on activation at +1.5 V for 60 s offered RSD of 6.7% (Figure 2a), while the biosensor built-up on activation at +1.7 V for 60 s offered RSD of 13.6% (Figure 2b).

#### 3.1.2. Modification of the Electrode by HSA

The other parameter which was optimized during the glycan biosensor preparation was the concentration of HSA applied for electrode modification. The results suggest that the ability of the glycan biosensor to detect DBA lectin increased with an increased concentration of HSA applied for electrode surface patterning almost in a linear fashion (Figure 2c). The highest sensitivity of −(2.30 ± 0.35) % M^−1^ (Figure 2c) for DBA detection was observed on the surface patterned by HSA from the 0.1 g L^−1^ HSA (~1.5 µM) solution. We did not test higher HSA concentration since we could expect formation of unwanted multilayers of HSA and this is why we considered 0.1 g L^−1^ HSA solution as the optimal one.

### 3.2. Characterization of the Surfaces

#### 3.2.1. Electrochemical Characterization of the Surfaces

**Figure 3:**
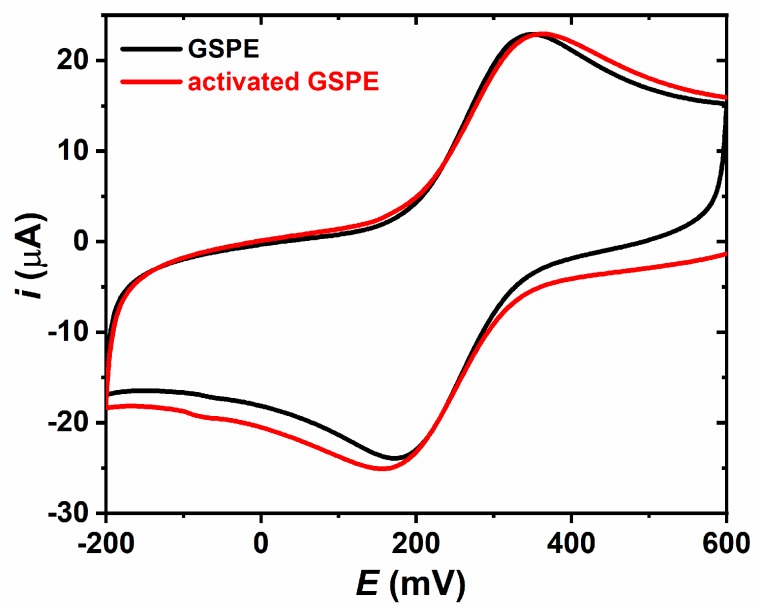
CV of 2.5 mM ferricyanide in PBS buffer on various electrodes assayed at a scan rate of 0.1 V s^−1^. Electrochemical surface area for GSPE is (0.149 ± 0.009) cm^2^ (roughness factor of 1.19) and for activated GSPE is (0.171 ± 0.010) cm^2^ (roughness factor of 1.36).

The GSPE and modified GSPE electrodes were characterized using cyclic voltammetry (Figure 3). The results indicate that at a scan rate of 0.1 V s^−1^, GSPE exhibits Δ*E* = (0.176 ± 0.033) V (Table S1), a value which increases to Δ*E* = (0.187 ± 0.032) V upon electrochemical surface activation (Table S2). The results thus confirm deposition of negative functional groups on GSPE during its electrochemical oxidation. From such electrochemical investigation we calculated also electrochemical surface area as follows: (0.149 ± 0.009) cm^2^ (roughness factor of 1.19) for GSPE and (0.171± 0.010) cm^2^ (roughness factor of 1.36) for activated GSPE. Covalent immobilization of HSA resulted in slight decrease of Δ*E* to a value of (0.177 ± 0.047) V (Table S3), a value, which then decreased to (0.156 ± 0.021) V after covalent attachment of the Tn antigen (Table S4).

#### 3.2.2. Raman Spectroscopy

**Figure 4:**
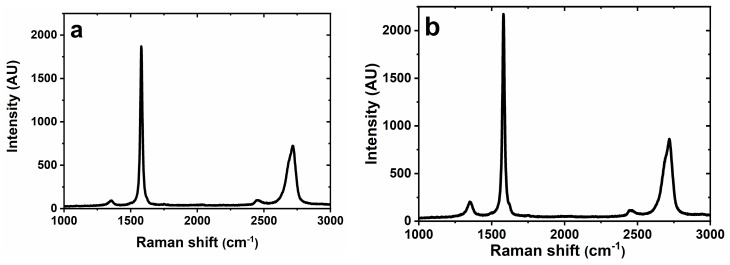
Raman spectra obtained for (**a**) plain graphene SPE and for (**b**) activated graphene SPE

Only two interfaces were characterized using Raman spectroscopy — GSPE before and after activation (Figure 4). The positions of the peaks at ~1350 cm^−1^ (D peak), ~1580 cm^−1^ (G peak) and ~2720 cm^−1^ (2D peak) are consistent with the literature [36,37] describing graphene-based screen-printed electrodes and peak at ~2450 cm^−1^ can be attributed to D+D´´ peak [38]. The position of the G peak at ~1580 cm^−1^ indicates the presence of multi-layered graphene or graphite [37]. The same conclusion can be judged from position of 2D peak at ~2720 cm^−1^, but small shoulder at ~2685 cm^−1^ indicated presence of two-layered or three-layered graphene flakes [39]. Presence of few-layered graphene flakes can be confirmed by low D/G value of 0.0495 (before activation) and 0.0944 (after activation) [40]. A positive shift of 2D peak from 2716 cm^−1^ (before activation) towards 2719 cm^−1^ (after activation) and a positive shift of G peak after activation (1580 cm^−1^ → 1581 cm^−1^) indicates that the sample after activation is more oxidized [41]. Also an increase of D/G ratio from 0.043 ± 0.010 to 0.080 ± 0.015 after activation indicates presence of oxidized species indicating presence of defects [32]. All data obtained from Raman spectroscopy are summarized in Table S5.

#### 3.2.3. Contact Angle Measurements

**Figure 5:**
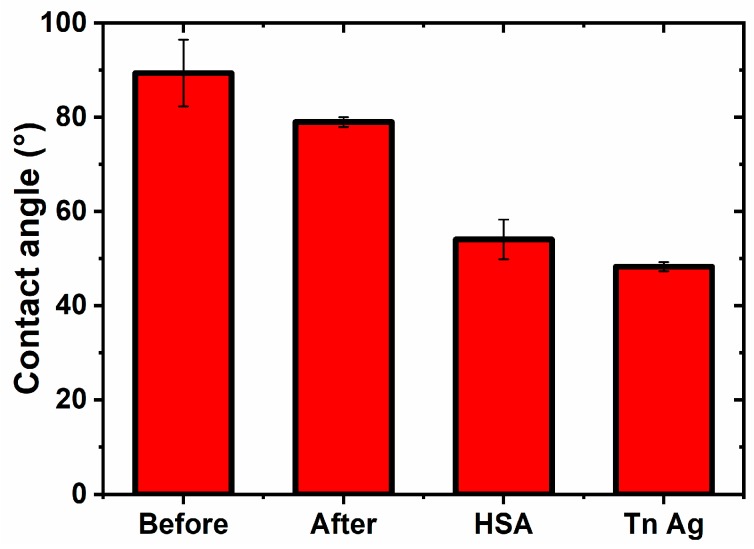
Contact angle measurements on various interfaces during preparation of the glycan biosensor.

The step-by-step built-up of the glycan biosensor can be monitored by changes in the contact angle measurements. Such analysis really confirmed that the highest contact angle was observed on GSPE (89.4 ± 7.1)°, and by subsequent surface patterning contact angle decreased to (79.0 ± 1.0)° (after anodic activation), (54.1 ± 4.1)° (after HSA immobilization) and finally reached the value of (48.3 ± 1.0)° for the surface with Tn antigen immobilized to it (Figure 5). Such assays confirmed the successful built-up of the biosensor interface. An increase of hydrophilicity of the graphene electrode by electrochemical oxidation is obvious due to delivery of oxygen containing groups. Upon incubation of the modified electrode with HSA further increase in hydrophilicity observed can be explained by delivery of high density of amino acids having a diverse range of functional groups and charges. Final incubation with Tn antigen further increases hydrophilicity of the surface by delivering high density of hydrophilic -OH groups.

#### 3.2.4. Scanning Electron Microscopy (SEM)

**Figure 6:**
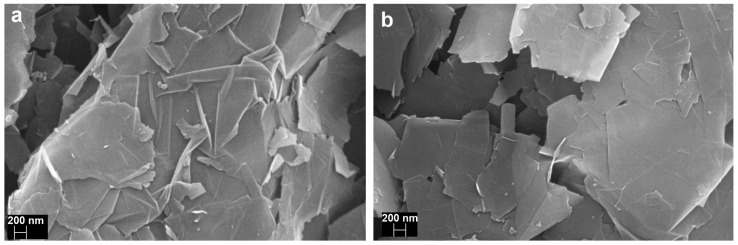

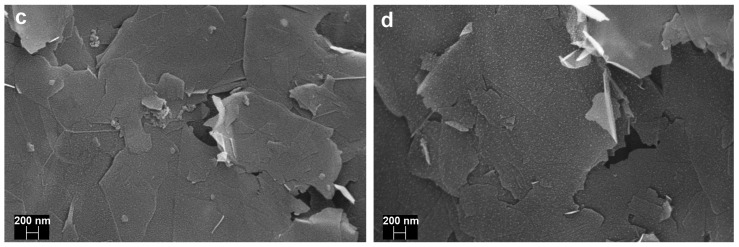
Scanning electron micrographs of (**a**) a plain graphene SPE, (**b**) an activated graphene SPE, (**c**) an electrode surface after HSA attachment and (**d**) an electrode after covalent immobilization of Tn antigen on the HSA layer. The scale in all SEM images is 200 nm.

SEM analysis confirmed presence of graphene flakes of different sizes re-stacked on the surface of GSPE (Figure 6a,b). SEM images revealed that the morphology of the interface after bioconjugation did not change (Figure 6c,d). More SEM images can be found in ESM file (Figures S2 and S3).

#### 3.2.5. EDX Measurements

EDX is able to provide information about the composition of quite thick interfacial layer (1–2 µm). The results obtained by this method suggest that a slight increase of carbon content was observed upon anodic activation of graphene SPE i.e., from a value of (88.1 ± 2.3)% to a value of (92.8 ± 1.7)%. At the same time, the oxygen content decreased from a value of (9.5 ± 1.2)% to a value of (6.7 ± 1.6)%. After HSA attachment, a significant N content was present on the surface i.e., (17.5 ± 0.7)% due to deposition of amino acids. After Tn antigen immobilization the N content slightly dropped with a mild increase of O content (see Table S6 in the ESM file). Thus we can conclude successful immobilization of both HSA and Tn antigen on the electrode surface. 

#### 3.2.6. Secondary Ion Mass Spectrometry (SIMS) Measurements

SIMS experiments confirmed successful immobilization of HSA molecules as a nanoscaffold on activated GPSE (Figure S4 *vs.* Figure S5). While SIMS spectra on activated GPSE with immobilized HSA confirm presence of several types of ion fragments containing N element (i.e., CH_4_N^+^, C_2_H_6_N^+^, C_3_H_8_N^+^, C_5_H_10_N^+^, C_11_H_24_N^+^, etc.) in a positive polarity (Figure S5), on activated GPSE such ion fragments were not identified (Figure S4). This indicated successful modification of activated GPSE by HSA molecules.

#### 3.2.7. Atomic Force Microscopy (AFM) Measurements

AFM experiments were run on activated GPSE electrode without any HSA immobilized and on the activated GPSE with HSA covalently attached on the activated GPSE. The results showed that on the interface with HSA immobilized there are present features having height of ~5.5 nm (Figure S6), what is in an excellent agreement with expected size of the HSA molecule. Therefore, HSA molecules were successfully attached on the interface.

#### 3.2.8. The Glycan Biosensor

**Figure 7:**
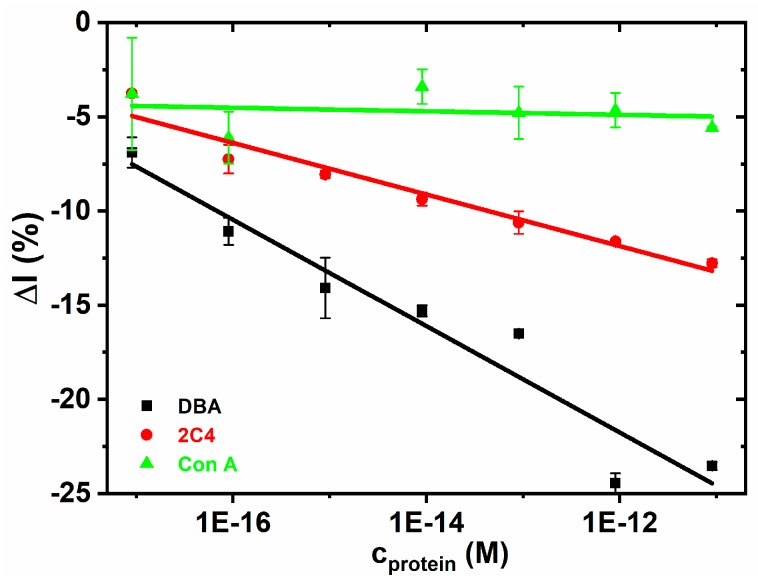
The binding preference of the Tn glycan biosensor was investigated using a negative control (Con A, a lectin not recognizing Tn antigen), a positive control (DBA lectin, recognizing Tn antigen) and an analyte (a tumor associated antibody 2C4). DPV was measured in an electrolyte containing 5 mM potassium hexacyanoferrate (II) trihydrate and 0.01 M PBS, pH 7.4. The parameters applied for the differential pulse voltammetry were as follows: 60 s accumulation time at 0.2 V, 50 ms modulation time, 0.5 s interval time, 25 mV modulation amplitude, and 5 mV step. Linear regression fit for DBA: Δ*I* = (−55.6 ± 4.8) + (−2.82 ± 0.34)×*c*, *R*^2^ = 0.918 and for the 2C4 antibody Δ*I* = (−28.3 ± 2.0) + (−1.37 ± 0.14)×*c*, *R*^2^ = 0.940. The plot for Con A could not be linearly fitted.

**Figure 8:**
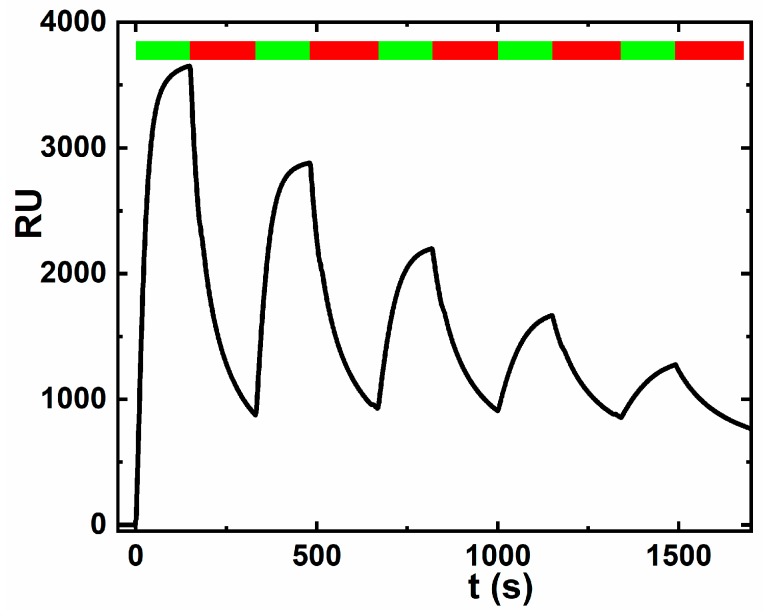
SPR experiment showing binding of 2C4 antibody towards the Tn antigen. Association phase is indicated by a green timeframe, while dissociation phase by a red timeframe as shown in the upper part of the figure. The SPR study was carried out with the following concentration of 2C4: 50 nM, 25 nM, 12.5 nM, 6.3 nM and 3.1 nM.

Finally, after optimization of the interfacial layer designs and its characterization, the glycan biosensor was constructed with the Tn antigen immobilized on a HSA-modified GSPE. The results indicated a negligible interaction for a negative control (Con A), significantly higher sensitivity of –(2.82 ± 0.34) % M^−1^ for a positive control (DBA, *R*^2^ = 0.918) and a high sensitivity of −(1.37 ± 0.14) % M^−1^ for the analyte (2C4, *R*^2^ = 0.940) (Figure 7). The results indicate that average non-specific binding is approximately 3.3% of the specific response towards DBA lectin and only 6.9% of the specific response towards the 2C4 analyte. The results obtained here are consistent with our previous report that DBA lectin is 1.3-fold better binder than 2C4 antibody towards Tn antigen immobilized on HSA on gold [35], but in case of immobilization of Tn antigen on HSA attached to GSPE, the sensitivity ratio DBA:2C4 is much higher i.e., 2.1-fold. Specific binding of 2C4 towards the Tn antigen was confirmed in the SPR study (Figure 8), while when using PNA (*Peanut* agglutinin) lectin as a control no binding was observed. PNA was in the SPR experiment applied as a control, since Con A would interfere with the dextran matrix of the CM5 SPR chip. Reproducibility of the glycan biosensor preparation is satisfactory within 10–12%. Detection limit for analysis of DBA and 2C4 is in low aM level (Table 1). 

Recently we summarized the applications of graphene-based electrochemical glycan biosensor for detection of a wide range of analytes including proteins, lectins, intact viruses and human liver cancer cells [22]. Some of these devices were built on graphene patterned interfaces using either field-effect transistor based biosensing [42] or a transducing scheme involving immobilized glycans with a built in redox moiety [43,44]. Such graphene-based glycan devices could detect analytes down to nM level, what means that our electrochemical glycan biosensor based on Tn antigen immobilized on HSA layer attached to graphene SPE is few orders of magnitude more sensitive compared to these devices [42,43,44] (Table 1). This suggests that quite high limit of detection obtained using devices previously published [42,43,44] is a result of detection platform applied rather than the attribute of graphene modified interface.

## 4. Conclusions

In this study we show that careful design of an interfacial layer on GSPE leads to the development of an ultrasensitive glycan biosensor able to detect its analyte — a tumor-associated antibody— down to low aM level. At the same time the glycan biosensor is able to selectively detect its analyte with only a minute response after addition of a control protein (Con A). Reproducibility of the biosensor preparation is satisfactory, within 10–12%. When compared with other electrochemical glycan based biosensors, our device is much more sensitive in terms of limit of detection achieved [42,43,44]. There is still a need to investigate robustness of the label-free assay system in an array format of analysis and to compare it with other commercially available label-free systems such as surface-plasmon resonance-based ones.

## Figures and Tables

**Table 1:** Performance of graphene-based electrochemical glycan biosensors.

Electrode	Glycan	Immobilization	Detection	Analyte	LOD	Ref.
CCG	pyrene-functionalized monosaccharides	π–π stacking interactions	FET sensing	Lectins	~1 nM	[42]
graphene	antraquinonyl-modified monosaccharides	π–π stacking interactions	DPV	Lectins	~1 µM	[43]
graphene SPE	pyrenyl-anthraquinone monosaccharides	π–π stacking interactions	DPV	Lectins	~50 nM	[44]
graphene SPE	pyrenyl-anthraquinone monosaccharides	π–π stacking interactions	DPV	Cells	~200-400 cells/mL	[44]
oxidized graphene	Tn antigen	covalent on HSA	DPV	Lectin (DBA)	~1 aM	This work
oxidized graphene	Tn antigen	covalent on HSA	DPV	Antibody (2C4)	~10 aM	This work

CCG—chemically converted graphene using hydrazine, DPV—differential pulse voltammetry, FET - field-effect transistor, LOD—limit of detection, SPE—screen-printed electrodes.

## Supplementary Materials

The following data are available online at https://www.mdpi.com/1424-8220/19/24/5409/s1, Figure S1: Binding of DBA on the glycan biosensor at higher concentration of DBA, Figure S2: SEM images of bare GSPE at different magnifications. Figure S3: SEM images of activated graphene SPE at different magnifications, Table S1: Basic electrochemical properties obtained using ferricyanide as a redox probe on GSPE., Table S2: Basic electrochemical properties obtained using ferricyanide as a redox probe on activated GSPE., Table S3: Basic electrochemical properties obtained using ferricyanide as a redox probe on activated GSPE with immobilized HSA., Table S4: Basic electrochemical properties obtained using ferricyanide as a redox probe on activated GSPE with immobilized HSA., Table S5: Characterization of plain GSPE before and after activation using Raman spectroscopy, Table S6: EDX analysis of various interfaces.
