# Supplementary material for: A Graphene-Based Glycan Biosensor for Electrochemical Label-Free Detection of a Tumor-Associated Antibody
Source: Sensors (Basel). 2019 Dec 9;19(24):5409. doi: 10.3390/s19245409 (PMC6960651; doi:10.3390/s19245409)
Supplement: Supplementary file 1 [file sensors-19-05409-s001.pdf]

SUPPORTING INFORMATION

A Graphene-Based Glycan Biosensor for Electrochemical Label-Free Detection of a Tumor-Associated Antibody

Filip Kveton [1], Anna Blsakova [1], Lenka Lorencova [1], Monika Jerigova [2,3], DusanVelic [2,3], Ola Blixt [4], Bo Jansson [5], Peter Kasak [6,*] and Jan Tkac [1,*]

1. Department of Glycobiotechnology, Institute of Chemistry, Slovak Academy of Sciences, Dubravska cesta 9, Bratislava 845 38, Slovakia; filipkvietok@gmail.com (F.K.); chemabls@savba.sk (A.B.); lenka.lorencova@savba.sk (L.L.)
2. Department of Physical Chemistry, Faculty of Natural Sciences, Comenius University, Mlynska Dolina, Bratislava 842 15, Slovakia; jerigova@ilc.sk (M.J.); duvellabs@gmail.com (D.V.)
3. International Laser Centre, Ilkovicova 3, Bratislava 841 04, Slovakia
4. Department of Chemistry, University of Copenhagen, 1871 Copenhagen, Denmark; olablixt@chem.ku.dk (O.B.)
5. Division of Oncology and Pathology, Department of Clinical Sciences, Lund, Lund University, Lund, SE 221 85, Sweden; bo.jansson@med.lu.se (B.J.)
6. Center for Advanced Materials, Qatar University, P.O. Box 2713, Doha, Qatar; peter.kasak@qu.edu.qa (P.K.)

* Correspondence: Jan.Tkac@savba.sk; Tel.: +421 2 5941 0263; Fax: +421 2 5941 0222; Peter.Kasak@qu.edu.qa; Tel.: +974 4403 5674.

EXPERIMENTAL SECTION

Scanning Electron Microscopy (SEM)

The morphology and homogeneity of bare and modified electrodes (after electrochemical activation, HSA and Tn immobilization) was investigated by SEM using an EVO 40HV apparatus (Carl Zeiss, Oberkochen, Germany).

Raman Spectroscopy

Raman spectra were measured with a DXR Raman Microscope (Thermo Scientific, Waltham, MA, USA) with 532 nm laser in the region from 3,500 to 52 cm^{-1} (laser power: 4 mW, exposure time: 5 s, number of exposures: 10, aperture: 50 μm pinhole, grating: 900 lines mm^{-1}).

Contact Angle Measurements

Contact angle measurements were run on a portable System E instrument (Advex Instruments, Brno, Czech) to reveal wetting angle for interfaces of bare and modified SPEs (after electrochemical activation, HSA and Tn immobilization). The volume of each droplet investigated was 20 μL and the testing liquid was DW. At least three contact angles were measured using three different droplets for each sample.

Energy-dispersive X-ray spectroscopy (EDX) measurements

EDX analysis (AXS, Bruker, Billerica, MA, USA) was used to determine the differences in chemical composition of bare and modified electrodes (after electrochemical activation, HSA and Tn antigen immobilization).

Secondary Ion Mass Spectrometry (SIMS)

SIMS is a technique for sensitive chemical surface analysis of samples. The analysis is not limited by the origin or type of a sample, that can be substantially any, inorganic, organic and biological. SIMS employing Time-of-Flight (TOF) analyzer provides elemental, chemical state and molecular information from surface layers or thin film structures with high sensitivity on the level of ppm-ppb. Besides, TOF SIMS IV spectrometers could provide high mass resolution, lateral resolution of 100 nm and a depth resolution of 1 nm. With the primary ion beam scanning across the sample surface, even the 2D chemical imaging of elements or molecules can be obtained providing data on a spatial distribution of predefined species. Mass spectrometry measurements were performed using a TOF-SIMS IV (ION-TOF, Muenster, Germany), a reflectron type of time-of-flight mass spectrometer equipped with a Bismuth ion source. Pulsed 25 keV Bi^+ were used as primary ions with ion current of 1 pA. The TOF-SIMS spectra were measured by scanning over the 100 μm × 100 μm analysis area with a total primary ion dose density below the static limit of 10^{13} ions cm^{-2}. SIMS images were measured by scanning over the 500 μm × 500 μm analysis area, with a lateral resolution of 5 μm. All assays were performed in a positive and a negative polarity.

Atomic force microscopy (AFM)

Atomic force microscopy measurements were applied to visualize and characterize interfacial features at the nanoscale. A Bioscope Catalyst instrument (Bruker, Billerica, MA, USA) and an IX71 microscope (Olympus, Bruker, Billerica, MA, USA) in conjunction with NanoScope 8.15 software were run at a scan rate of 0.5 line s^{-1} with the tip set to 200 pN (Scan Asyst, Bruker, Billerica, MA, USA). Visualizations were made using quantitative nanomechanical mapping in air. Bare and HSA modified SPCE graphene electrodes (Metrohm DropSens, Asturias, Spain) were prepared for analysis, as described previously. The SCANASYSTAIR silicon tip on a nitride lever (Bruker, with f_0 = 50–90 kHz and k = 0.4 Nm^{-1}), sharpened to a tip radius of 2 nm, was used in the measurements.

Surface Plasmon Resonance (SPR) experiments

HBS-P+ (containing 0.1 M HEPES, 1.5 M NaCl and 0.5% v/v Surfactant P20, pH 7.4 after dilution 10×) from GE Healthcare (Uppsala, Sweden) was used as a running buffer. SPR measurements were run at Biacore X100 instrument (GE Healthcare, Milwaukee, WI, USA). Sensor chip CM5 used for Tn-BSA immobilization and subsequent kinetic analysis study was also purchased from GE Healthcare (Uppsalaป Sweden). Ethanolamine hydrochloride obtained from GE Healthcare (Sweden) was used as a blocking agent. Elution solution ES-2100 used for regeneration of sensor chip surface was obtained from Vector Laboratories (Burlingame, CA, USA).

All SPR experiments were performed using a dual channel Biacore X100 SPR system controlled by the SPR Biacore control software. Immobilization of Tn antigen conjugated to BSA (Tn-BSA) as ligand to CM5 sensor chip was performed at the flow rate of 5 μL min^{-1}. Amine coupling was employed for immobilization of Tn-BSA. The carboxylic groups of CM5 chip dextran matrix were activated with freshly prepared mixture of 0.2 M EDC and 0.05 M NHS in ratio 1:1. After this step, the Tn-BSA was injected for 10 min. The concentration of Tn-BSA applied for immobilization was 50 μg mL^{-1} in a 10 mM sodium acetate buffer pH 4.0. The contact time was set up to 420 s during the immobilization process. After immobilization of Tn-BSA was completed, 1 M ethanolamine hydrochloride was added for 10 min in an effort to deactivate free carboxylic groups and decrease nonspecific interactions. The surface of CM5 chip was regenerated with 20 mM NaOH.

Kinetic analysis measurements were performed for 2C4 (analyte) and for PNA (*Peanut* agglutinin) lectin. PNA was in the SPR experiment applied as a control, since Con A would interfere with the dextran matrix of the CM5 SPR chip. In this kinetic study the proteins were injected at the flow rate of 30 μL min^{-1}. The CM5 chip was regenerated by 30 s pulse of ES-2100 elution and then the surface was regenerated with 20 mM NaOH. HBS-P+ was used as a running buffer for dilutions. All the measurements were performed at room temperature (25 °C).

The measured data were fitted by Biacore evaluation software with configuration 1:1 binding. After fitting we obtained kinetic parameters such as association and dissociation constants as mentioned below. SPR sensorgram represents real-time dependence of response unit *vs.* time.

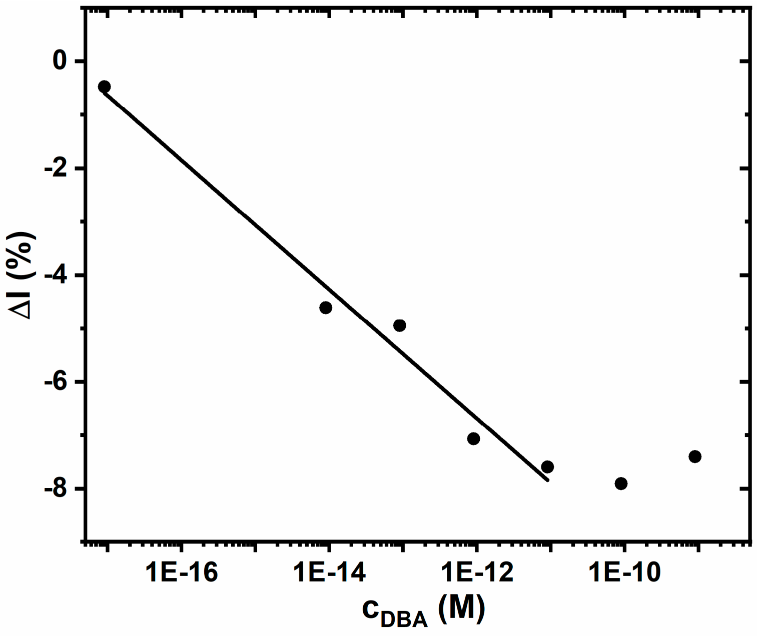

Figure S1. Binding of DBA to the Tn glycan biosensor investigated in the concentration window from 9.10^{-18} up to 9.10^{-9} M. DPV was measured in an electrolyte containing 5 mM potassium hexacyanoferrate (II) trihydrate and 0.01 M PBS, pH 7.4. The parameters applied for the differential pulse voltammetry were as follows: 60 s accumulation time at 0.2 V, 50 ms modulation time, 0.5 s interval time, 25 mV modulation amplitude, and 5 mV step.

Table S1. Basic electrochemical properties obtained using ferricyanide as a redox probe on GSPE.

Scan rate / V s^{-1}	E_{pc} / V	E_{pa} / V	ΔE / V	i_{pc} / μA	i_{pa} / μA	i_{pc} / i_{pa}
0.1	0.173	0.350	0.176	−23.9	22.9	−1.045
0.2	0.148	0.373	0.225	−35.2	35.2	−1.000
0.3	0.131	0.385	0.254	−44.8	45.5	−0.984
0.4	0.120	0.402	0.283	−53.4	54.8	−0.974
0.5	0.105	0.417	0.313	−61.5	62.7	−0.980
0.6	0.087	0.429	0.342	−69.3	71.0	−0.976
0.7	0.078	0.444	0.366	−76.7	79.1	−0.970
0.8	0.068	0.454	0.386	−84.0	86.7	−0.969
0.9	0.036	0.473	0.437	−91.1	94.1	−0.969

Table S2. Basic electrochemical properties obtained using ferricyanide as a redox probe on activated GSPE.

Scan rate / V s^{-1}	E_{pc} / V	E_{pa} / V	ΔE / V	i_{pc} / μA	i_{pa} / μA	i_{pc} / i_{pa}
0.1	0.173	0.360	0.187	−26.4	24.7	−1.073
0.2	0.144	0.385	0.242	−39.3	38.1	−1.032
0.3	0.119	0.406	0.287	−50.2	49.1	−1.025
0.4	0.092	0.426	0.333	−60.2	59.1	−1.020
0.5	0.065	0.443	0.377	−69.6	68.0	−1.024
0.6	0.037	0.460	0.422	−78.5	76.8	−1.024
0.7	0.017	0.473	0.457	−87.3	84.2	−1.037
0.8	0.016	0.500	0.516	−95.3	92.0	−1.037

Table S3. Basic electrochemical properties obtained using ferricyanide as a redox probe on activated GSPE with immobilized HSA.

Scan rate/Vs^{-1}	E_{pc}/V	E_{pa}/V	ΔE/V	i_{pc}/μA	i_{pa}/μA	i_{pc}/i_{pa}
0.1	0.170	0.347	0.177	−41.1	39.6	−1.037
0.2	0.145	0.373	0.228	−61.7	60.7	−1.016
0.3	0.124	0.395	0.271	−79.1	78.3	−1.010
0.4	0.103	0.413	0.310	−95.1	94.2	−1.010
0.5	0.083	0.430	0.347	−110.0	109.2	−1.008
0.6	0.066	0.447	0.381	−124.1	123.3	−1.006
0.7	0.049	0.464	0.415	−137.8	136.8	−1.007
0.8	0.030	0.479	0.449	−150.5	149.8	−1.005
0.9	0.010	0.492	0.482	−163.0	162.2	−1.005

Table S4. Basic electrochemical properties obtained using ferricyanide as a redox probe on activated GSPE with immobilized HSA.

Scan rate/V s^{-1}	E_{pc}/V	E_{pa}/V	ΔE/V	i_{pc}/μA	i_{pa}/μA	i_{pc}/i_{pa}
0.1	0.182	0.339	0.156	−41.4	40.5	−1.023
0.2	0.164	0.363	0.199	−61.2	60.3	−1.014
0.3	0.141	0.374	0.233	−78.3	76.8	−1.020
0.4	0.123	0.398	0.276	−93.4	91.2	−1.023
0.5	0.108	0.428	0.320	−107.5	105.2	−1.022
0.6	0.088	0.447	0.359	−120.9	118.4	−1.021
0.7	0.075	0.458	0.383	−133.8	131.1	−1.020
0.8	0.057	0.475	0.419	−146.0	143.2	−1.019
0.9	0.049	0.489	0.439	−157.6	154.6	−1.019

Table S5. Characterization of plain GSPE before and after activation using Raman spectroscopy.

	Before activation		After activation	
	**Position (cm^{-1})**	**Intensity (a. u.)**	**Position (cm^{-1})**	**Intensity (a. u.)**
D	1349	92.6	1349	205
G	1580	1869	1581	2172
D + D′′	2449	98.8	2448	114
2D	2716	722	2719	862
D/G		0.0495		0.0944
2D/G		0.386		0.397

Table S6. EDX analysis of various interfaces.

	Bare GSPE		Activated Electrode		HSA attachment		Tn immobilization	
	**Average at%**	**SD %**	**Average at%**	**SD %**	**Average at%**	**SD %**	**Average at%**	**SD %**
C	88.1	2.3	92.8	1.7	75.1	1.6	77.4	4.8
N	-	-			17.5	0.7	15.3	4.8
O	9.5	1.2	6.7	1.6	5.2	0.4	6.4	0.6

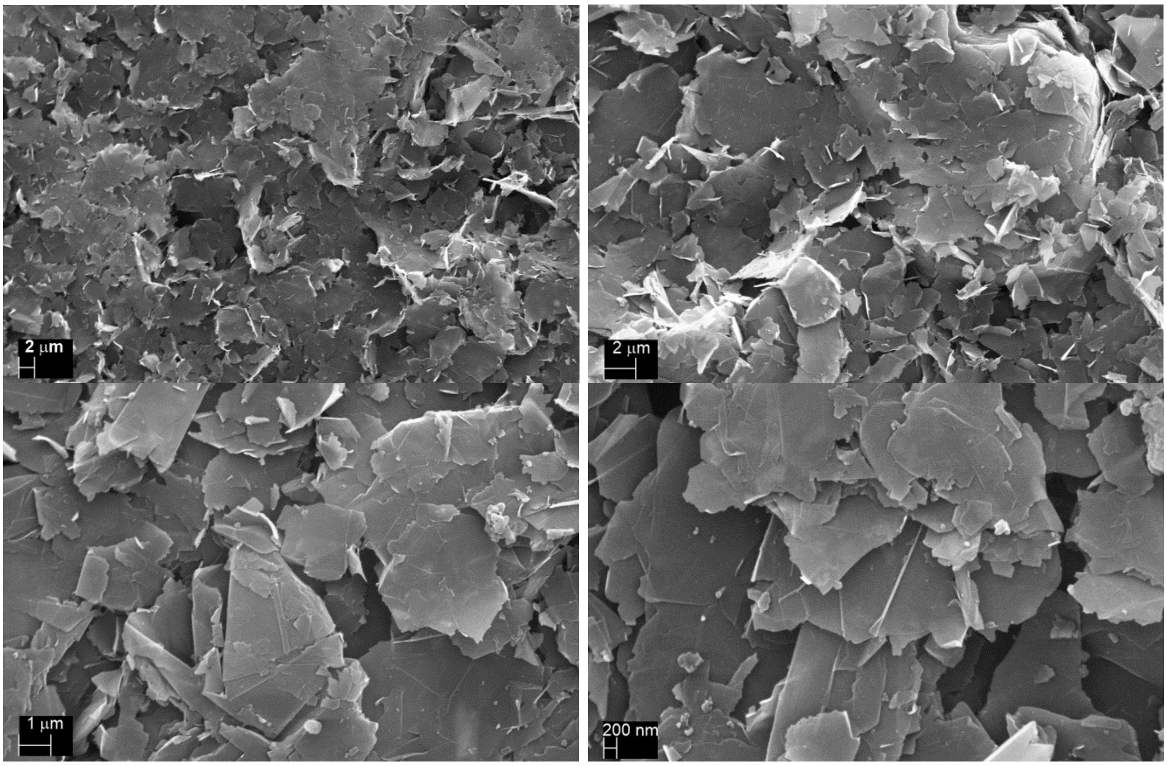

Figure S2. SEM images of bare GSPE at different magnifications.

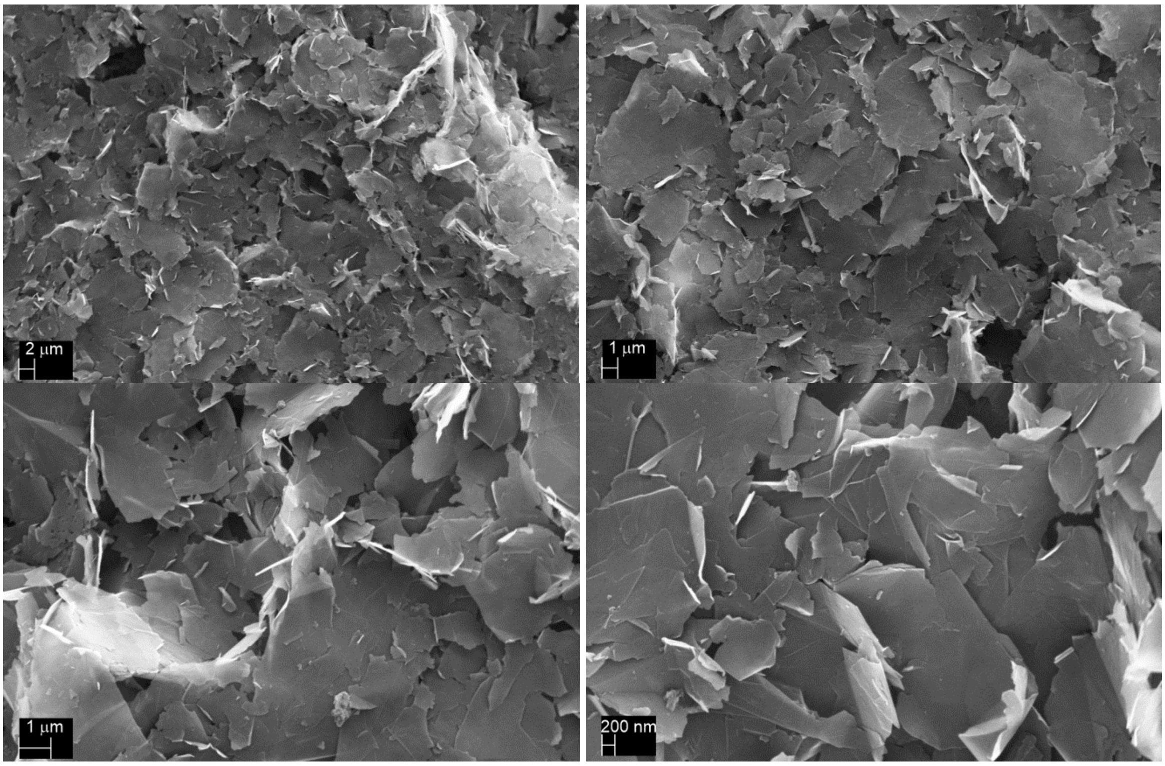

Figure S3. SEM images of activated graphene SPE at different magnifications.

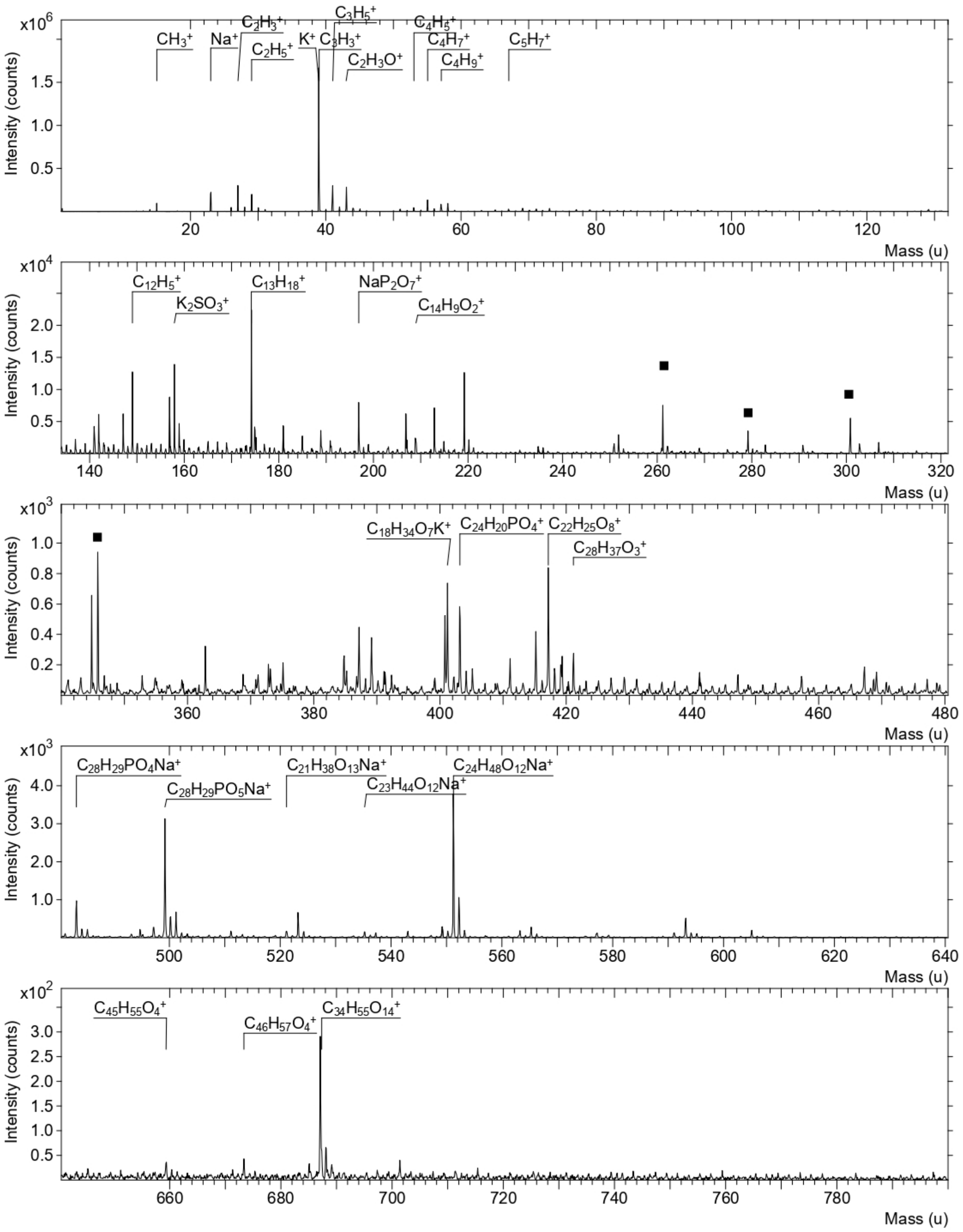

Figure S4. SIMS spectra for fragments of activated graphene SPE in a positive polarity.

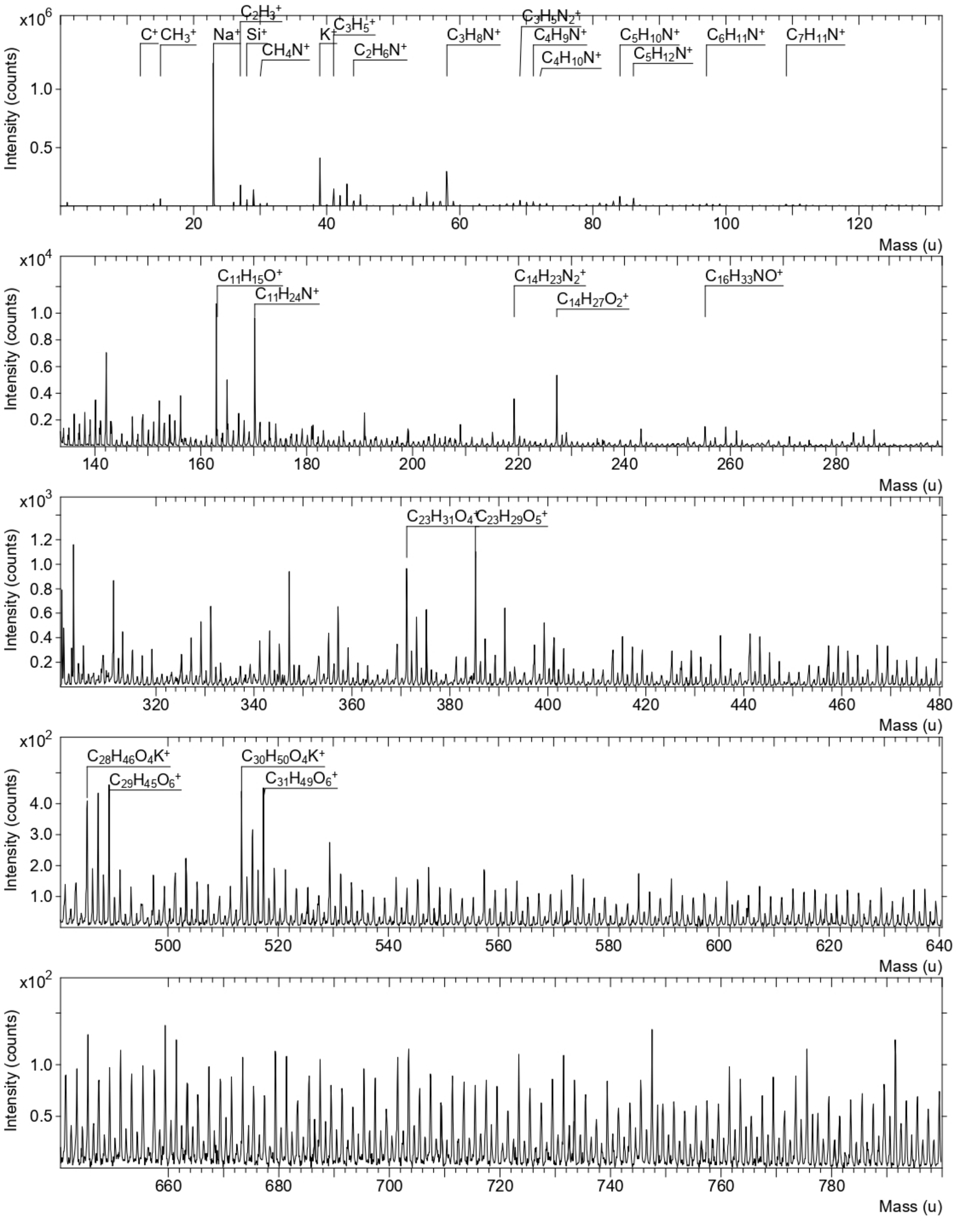

Figure S5. SIMS spectra for fragments of activated graphene SPE with immobilized HSA in a positive polarity.

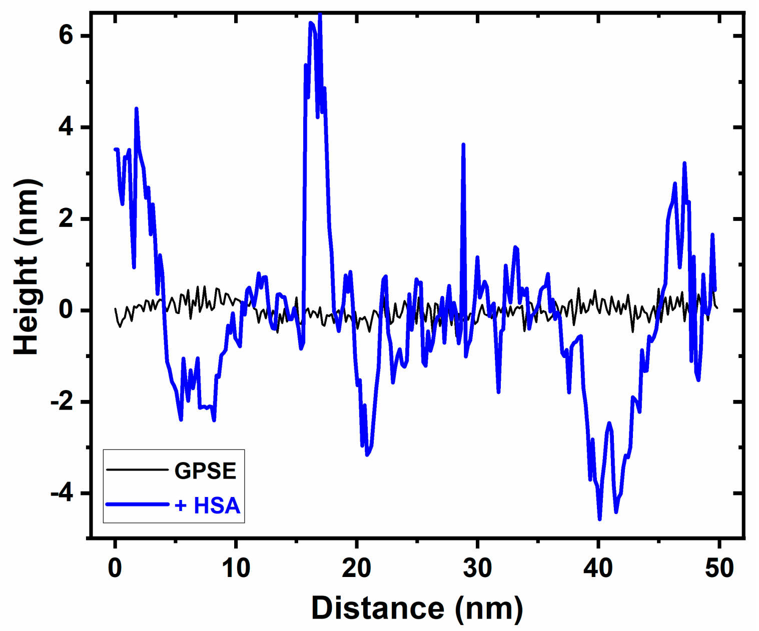

Figure S6. AFM profile for GPSE and GPSE with covalently immobilized HSA.
